# Translating the Patient Perception of Integrated Care Survey to Measure Integrated Care in the Netherlands: Combining Equivalence and Contextualization Approaches for Optimal Results

**DOI:** 10.5334/ijic.2022

**Published:** 2016-08-23

**Authors:** Maike V. Tietschert, Federica Angeli, Arno J. A. van Raak, Sara J. Singer, Dirk Ruwaard

**Affiliations:** 1 Maastricht University, Department of Health Services Research, Room 0.043, Duboisdomein 30, 6229 GT Maastricht, The Netherlands; 2 Maastricht University, Department of Health Services Research, Room 0.073, Duboisdomein 30, 6229 GT Maastricht, The Netherlands; 3 Maastricht University, Department of Health, Services Research, Room 0.063, Duboisdomein 30, 6229 GT Maastricht, The Netherlands; 4 Harvard T. H. Chan School of Public Health, Department of Health Policy and Management, 677 Huntington Avenue Kresge, Room 407, Boston, Massachusetts 02115, USA; 5 Maastricht University, Department of Health Services Research, Room 0.083, Duboisdomein 30, 6229 GT Maastricht, The Netherlands

**Keywords:** integrated care, survey translation, translation method, patient perception, cross-cultural

## Abstract

**Introduction:**

An increase in initiatives to improve integration of care provides the need for instruments that assess the degree of integrated care as perceived by patients across cultural contexts. This article aims to explain the relevance of equivalence and contextualization approaches in translating and adapting the Patient Perception of Integrated Care Survey developed in the US for use in the Netherlands.

**Theory and methods:**

The World Health Organization guidelines guided the translation and adaptation, including a forward-backward translation and patient-feedback through informal contacts (N4) and cognitive interviews (N14).

**Results:**

The forward-backward translation produced a Dutch version of the Patient Perception of Integrated Care Survey with minor adaptations. Patients evaluated the survey as very relevant. Alterations resulted from structural and cultural differences and specificities of patients with chronic conditions.

**Conclusions and discussion:**

A context-sensitive translation process is key to developing instruments for cross-cultural health research. Our results show that equivalence- and contextualization methods provide equally relevant, yet substantially different contributions to the translation outcome and should both be incorporated when translating instruments for different cultural contexts. The results support the applicability of the Patient Perception of Integrated Care Survey in the Netherlands and are promising for its adoption in other cultural contexts.

## Introduction

“Meeting the complex needs of patients with chronic illness or impairment is the single greatest challenge facing organized medical practice” [1, p. 2].

Chronic patients’ needs require multiple services and, due to specialization and professionalization of different occupational groups [2], cannot be addressed by one professional alone [3]. Integration of healthcare services and providers has become indispensable [4], giving rise to a number of initiatives all over the world that aim to improve the degree to which professionals integrate care [5,6,7,8].

Although of critical importance for practice and policy makers alike, little is known about which approaches actually improve various aspects of care integration [6,9]. Learning from on-going initiatives requires thorough evaluations and international comparison [8]. Yet, instruments that assess integrated care comprehensively are scarce. A review performed by Lyngsø et al. [10], although limited to provider perceived level of integrated care, could not identify any instruments assessing integrated care that are cross-culturally applicable. Research in other areas of health services research has shown that, although beneficial, translating instruments for use in different contexts and countries can have implications for the data quality and reliability [8]. This is particularly relevant to cross-cultural studies of integrated care, because the values and social norms underlying integrated care are shaped within different cultural contexts, which is expected to bear an influence on individuals’ perception of care delivery [11,12,13]. Hence, translating and adapting patient-reported measures to another cultural context requires a careful approach.

To facilitate valid translations and adaptations of surveys for cross-cultural use, translation studies have introduced different methods. At present, there is little agreement on which type of translation should be used – and how – to produce the most rigorous output [14]. Studies use different techniques and guidelines, which are mostly based on established practices rather than empirical results [15]. Limited empirical evidence is reported about what each method contributes to the translation [13,15,16]. Consequently, cross-cultural applicability of instruments is limited.

The pressing need for more empirically based insights on how best to perform cross-cultural translation combined with the need for cross-culturally applicable measures of integrated care requires addressing “challenges of replicating measurement instruments across different health care settings, guidelines on how best to develop measurement instruments that can more effectively be replicated in the health system of other countries and further validation and development of already existing measurement (p. 13)” [10]. Therefore, this paper addresses the following research question: How should different approaches for translations and cultural-adaptations be used for the translation of surveys that assess integrated care in different cultural contexts?

To do so, this paper describes the translation and adaptation process of the Patient Perception of Integrated Care (PPIC) Survey [9] for use in a research project that assesses integrated care in the Netherlands. The PPIC survey is developed by Singer and colleagues [9] in the US, based on two premises: First is that the degree to which integrated care is provided must be assessed independently from its organizational antecedents, which is why this survey is particularly suitable for cross-cultural use. Second is that the patient’s perspective should be the point of departure. The PPIC operationalizes the following definition of integrated care:

“Patient care that is coordinated across professionals, facilities, and support systems; continuous over time and between visits; tailored to the patients’ (and family members’) needs and preferences; and based on shared responsibility between patient and caregivers for optimizing health.” [17, p. 113].

This definition is further specified in a conceptual model, which describes integrated care along seven dimensions: coordination within, and across care teams, coordination between care teams and community resources, familiarity with patient over time, proactive and responsive action between visits, shared responsibility, and patient-centeredness [9,17]. That the framework underlying the PPIC indeed reflects patients’ understanding of integrated care is supported by the study of Walker et al. [6]. The authors conducted a series of focus groups with a diverse patient sample to explore patients’ perception of integrated care. The themes they discovered were consistent with the dimensions underlying the PPIC. So far, the PPIC has only been used in the American health care context for which first psychometric tests support reliability and validity [9].

To translate and culturally adapt the PPIC survey to the Dutch context, we apply techniques from two basic approaches and compare their separate contribution to the translation output. Thus, by accomplishing the translation of the PPIC survey for use in the Netherlands, this study contributes insights that can help to inform future translations and adaptations of health-related surveys for different cultural settings. We proceed by explaining two different translation approaches, the translation process that we followed and how this processes contributed to the translation outcome.

## Theory and Method

The literature describes two basic approaches to translation, namely equivalence and contextualization. According to the equivalence approach the aim of a translation is ‘to achieve a text in the target language that is equivalent, meaning having equal value, to the original source-language version’ [16, p. 563]. Studies seeking this objective predominately use techniques that ensure accuracy, validity, and reliability, such as forward-backward or team/committee translations. When seeking contextualization, “the translation is a form of intercultural interaction, rather than a lexical transfer of meaning” [16, p. 573]. This form of translation employs a hermeneutic and interpretive activity, which achieves quality if the original meaning is transmitted in a culturally adequate way. The idea underlying this approach is that instruments should not only receive adequate linguistic translation but also should adjust for cultural specificities of the new context to maintain content validity [11]. An example is provided by the study of Li, Wang and Shen [18] in which they translated the US-developed SF-36 Health Survey for use in China. To adapt an item that measured physical activity for the Chinese context they used Tai Chi as a complementary prompt because the original suggestions golf and bowling were not regular sports in China. Although linguistically these adaptations change the question, they helped to increase comparability of the construct that the item measured.

Along these same lines, Johnson [14] distinguishes between shared method and shared meaning. Shared method addresses equivalence and concerns technical problems of cross-cultural measurement, such as semantic and instrument equivalence or psychometric properties. Shared meaning follows the contextualization paradigm and aims for interpretive equivalence, being equivalence of the meaning of measures. These two approaches (equivalence and contextualization) formed the framework that guided our translation process. To translate the PPIC survey we started by following the equivalence approach. We then proceeded with contextualization methods to culturally adapt the PPIC survey to the target context. We will continue by describing the properties of the PPIC survey, followed by the translation process that we applied to translate and adapt the survey for use in the Netherlands to assess integrated care as perceived by patients of primary care centres.

### Measure

The PPIC survey (version 2.1) measures patients’ perceptions of integrated care for application in the US and served as basis for the translation and adaption process. The survey was theoretically derived and developed through multiple rounds of pilot-testing, cognitive testing, and input from an advisory panel of survey measurement and care integration experts, patient representatives, and patients [9,19]. Designed particularly for administration to patients with multiple and complex healthcare needs, the survey asks about patients’ experience of care across settings, including their primary provider’s office, specialists, hospitals, and at home, and over time. Reliability and validity were previously established in a US based sample.

### Development process

**Figure 1:**
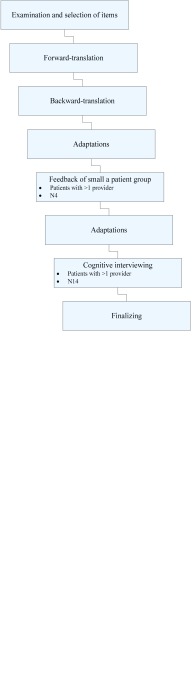
Translation process based on WHO guidelines.

In translating the survey, we followed the guidelines of the World Health Organization for translating questionnaires [20]. Figure 1 provides an overview of our translation process, which started in November 2013 and was finalized in August 2014.

We started by assessing the item applicability for our study purpose and target population. We included all items that measure the degree to which integrated care is provided. Additionally we included questions about the following demographics: general health status, age, gender, highest level of school, country of origin and whether somebody helped to complete the survey. In choosing our methodology we sought to achieve interpretative comparability, i.e., equality with which measures are interpreted across cultures (equality of meaning) and procedural comparability, which relates to technical problems of cross-cultural measurement (e.g. equality of items, measurement unit, or psychometric characteristics) [14]. We started with the equivalence approach in performing a forward-backward translation. Forward-backward translation was chosen because we aimed to produce a cross-culturally comparable survey for which this method is most suitable as it allows for direct comparison of the translated measure with the original [21]. The research team consisted of four researchers, fluent in English and Dutch. Two researchers are native Dutch, one German and one Italian. The forward translation was performed by the German and one Dutch researcher and then verified by the other researchers independently. Deviations and adjustments were discussed during group sessions. The developer of the PPIC survey verified the intended interpretation of questions in case of disagreement. After reaching consensus, a backward translation was requested from an independent translator at the University’s Language Centre. The backward translation was compared to the original and Dutch version. Mismatches were discussed among the research team and the translator until consensus was reached.

Applying forward-backward translation ensures comparability of the translated measure with the original measure even in case of poor translations, because the translated items may use the same structure as the translation and hence may perform well in the backward translation. Yet, it may not necessarily produce a translation that is appropriate for use in the target culture [21]. Although items might be semantically equivalent, their interpretation can vary across different cultural contexts. We therefore supplemented equivalence methods with contextualization 1) to ensure understandability of our survey and 2) to verify that items were measuring the constructs they were intended to measure. Because involving informants from the target population in the translation process is associated with increased user satisfaction and response completeness [22], we presented the survey to four respondents that were patients of a primary care centre in South Limburg, the Netherlands. Respondents were asked for feedback on understandability and relevance of each survey item.

Insights from the forward-backward translation and initial patient feedback were incorporated in a pilot version of the Dutch PPIC survey. To assess applicability and understandability of this version we then performed cognitive interviewing in group- and individual settings. Group interviews were performed because they allow comparison among respondents’ experiences and perceptions. Furthermore, group interviews facilitate interaction between respondents and can illuminate similarities and differences in reference frames that respondents apply when interpreting survey items [23]. Next, we performed individual interviews because, although group interviews offer the benefit of interaction, individual interviews are better suited to gain in-depth knowledge as they allow for direct probing of respondent’s knowledge [23]. Also, individual interviews allowed the inclusion of informants that were not able to travel to participate in group interviews due to health challenges. Inclusion of this group was very important because patients with complex health needs are likely to strongly benefit from receiving integrated patient care and hence are an important target audience [17]. The WHO guidelines advise testing each instrument section on at least ten patients [20]. We reached saturation after interviewing 14 patients (one group interview with four respondents, three interviews with pairs and four individual interviews). During the interview, the interviewer read the questions out loud. Respondents were asked whether questions were formulated clearly, to rephrase questions, explain what the items were asking and what they thought of when providing an answer. For difficult items, respondents were encouraged to provide suggestions for improvement. At the end of the interview, respondents were asked what they thought of the survey, whether the items cover their care experience and whether questions were missing. Each interview was recorded after respondents’ permission was gained. Insights from the interviews were used to finalize the survey. Finally, adapted questions were translated into English and presented to the developer of the PPIC survey to ask for feedback and approval (the survey is available from the authors on request). This study was exempt from review by the Medical Research and Ethics Committee since it was not liable according to the Dutch Medical Research (Human Subjects) Act [24].

### Study Population – cognitive interviewing

As the survey assesses inter-provider collaboration, respondents eligible for inclusion had to be seen by more than one health care provider in the previous six months. Respondents were approached via a primary care centre and an interest group for health care users in South Limburg, the Netherlands. Men and women were equally represented with ages ranging from 40 to 83.

### Data-analysis

The feedback from the cognitive interviews was summarized after the interviews were performed. To determine whether questions needed refinement the notes were reviewed to find similarities and differences in the respondents’ feedback. The findings and possible alterations of items were discussed within the research team.

## Results

### Forward-backward translation

After the forward translation was performed some questions needed shortening, because grammatical differences in the Dutch language resulted in over-complicated sentence structures. To ensure that question content was not affected, we checked for conceptual equivalence after the backward translation was performed. Alterations after the backward translation were minor and considered lexical changes, where synonyms were matched to the original version precisely. At this stage, we changed words in three items: ‘thoughts’ into ‘idea’, ‘good’ into ‘easy’ and ‘care’ into ‘instructions’. We also adapted three questions for which the introduction differed from the other items. Most questions in the survey begin with ‘In the last 6 month…’. However, questions about specialist care outside the provider’s office start with ‘In general’ but were erroneously translated with ‘in the last 6 months’. We also adapted one question where the word ‘sometimes’ was accidentally translated into ‘often’.

### First response by the initial four patients

We presented the survey to four patients to ask for their first impression on understandability and relevance of survey items before finalizing our survey for cognitive interviews. Respondents experienced difficulties in answering some questions that assess the provision of information and support because they did not feel they needed such services. For example, patients with chronic disease found it difficult to relate to the following question:

“In the last 6 months, how often have you and anyone of the primary care centre talked about how you were supposed to take your medicine?”

Respondents explained that they did not discuss how to take the medicine with their provider because they had been taking this medication for years. Hence, they perceived that this question was not relevant to their situation. The same applied to patients with non-complex health needs, in relation to the following question:

“In the past 6 months, how often did these other staff talk with you about care you received from your GP?”

Respondents struggled to answer this question because answering ‘never’ indicates that the staff did not provide integrated care, while instead they perceived that there was no need. Because the survey was presented to a small number of patients who may not cover all characteristics of the target population, we did not change any survey items at this stage. Instead, we added sub-questions that assessed patients’ needs for the type of care that the particular question was addressing to improve the interpretability of these items and to account for the heterogeneous population. These additions were verified during cognitive interviews.

### Cognitive interviewing

Respondents’ feedback for the applicability of the PPIC survey was positive. Items were evaluated as highly relevant and covering crucial aspects of health care delivery. However, respondents also identified possibilities for improvements. Below, we describe the main issues patients raised and how we addressed them.

Respondents experienced difficulties with questions that were referring to the content of the previous question, such as the case for the following two items:

Item 18: “In the last 6 months, did this provider talk with you about setting goals for your health?”

Item 19: “In the last 6 months, did the care you received from this provider help you meet your goals?”

It was not clear that the goals in question 19 were referring to the goals in question 18. To improve visual guidance and to reduce item length we subordinated referring questions and took out redundant repetitions. For example question 18 was changed to 17a and ‘your goals’ adapted to ‘these goals’.

Questions that asked about ‘instructions’ that providers advised the patient to follow were experienced as somewhat patronizing. Respondents explained that doctors can only give advice and that it is up to the patient to decide how they use it. We therefore added the word ‘advice’.

Furthermore, patients struggled with the item that asks whether the GP discussed setting goals for their health. Patients stated that the only goal of a patient is to get perfectly healthy but that this was not possible for most of them. Providing examples about other goals such as increasing physical activity or a healthier diet clarified the item but respondents expressed a discrepancy between these examples and their initial understanding of the question. However, possible goals could be many, and we did not want to limit the item to a set of examples. To provide more guidance to the respondent we added ‘setting goals to improve your health, maintain your health, or to slow down deterioration’ to the question.

Patients had difficulties in answering questions about the GP’s knowledge of patients’ medical history. According to respondents providers were well informed because they access information via the medical information system when seeing the patient. Respondents were satisfied with this approach but had problems answering the question, as strictly speaking the providers did not ‘know’ the information but had ‘consulted’ their medical record to access it. To overcome this problem we added consult to this question: ‘In the last 6 months, how often did this provider seem to know or consult the important information about your medical history?’

The item asking whether the patient had to contact the provider’s office him/herself to get the results of a medical test also required adaptation. In the Netherlands, it is normal procedure for patients to contact the provider’s office themselves. All respondents reported that this was the case. Respondents did not experience this as onerous as long as they were informed about it. Hence, we added the following question:

“When you had to contact the primary care centre yourself: Did they explain to you in advance that you had to contact them yourself?”

Other items requiring discussion were asking about contact with the primary care provider’s office outside regular office hours. Respondents stated that these questions were not applicable to the Dutch system, because patients either are redirected to an answer machine with information on where to go or are immediately redirected to the general practice service (huisartsenpost), a service for acute and urgent care needs outside regular office hours. This is a widely used system in the Netherlands of which patients are aware. Patients explained that they would not contact their provider outside office hours, because they know they have to contact the general practice service. Although this system is much institutionalized we consider it important to determine whether primary care centres ensure continuity of care. For this purpose we changed the following question from:

“In the last 6 months, when you tried to contact this provider’s office after regular office hours, how often did you get an answer to your medical question in a timely manner?”

to

“Did the primary care centre make sure that you knew where to go outside regular office hours?”

Respondents had difficulties providing one overall score for the care they received because there are large differences in the quality of care they receive from different providers, which they claimed makes it impossible to weigh these differences in one score. To prevent respondents from skipping this item we separated answer categories from an overall score to individual scores for each provider group in the survey (GP, other staff of the provider’s office and the specialist).

We also asked patients whether the survey misses aspects that are important to their care delivery. An item that was missing concerned the evaluation of medicine. Patients explained that their GP, when prescribing new medicine, typically thoroughly explained how to take this medicine, but may not have evaluated whether the current medication intake was still up to date. Patients described situations where they felt nauseous for long periods because the medication intake did not meet their needs anymore. We therefore added the following question:

“In the last 6 month: did somebody from the primary care centre look at your medication intake with you?”

## Discussion

This paper describes the approach used to translate and culturally adapt the Patient Perception of Integrated Care (PPIC) survey for use in the Dutch health care sector. The WHO guidelines proved to be very useful in guiding this process. Using both equivalence and contextualization approaches was particularly valuable, as each step of the process led to significant improvements in the applicability of the survey for the Dutch context.

**Figure 2:**
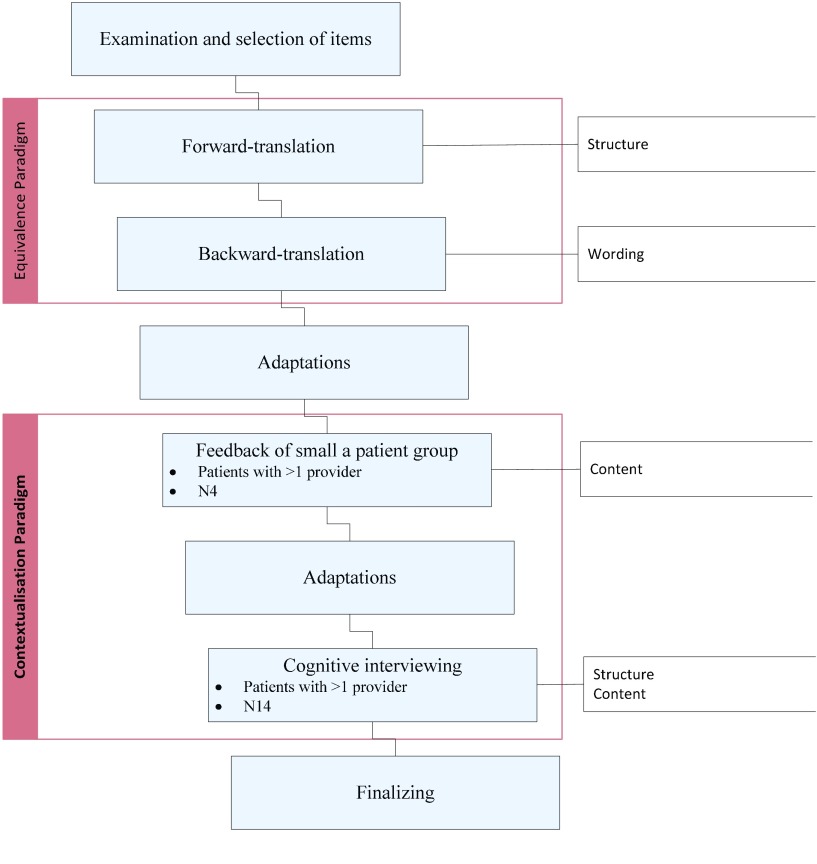
Translation process outcomes.

Figure 2 highlights how both methods provided different contributions to the adaptations process. Methodologies that belong to the equivalence approach revealed the need for lexical and formal adaptations. The forward-backward translation process showed that choosing adequate wording is a delicate process and needs a thorough examination. This is particularly relevant for words that have several synonyms in the target language that, depending on the context, one or the other of which may better represent the source wording. Formal adaptations were needed because some items were lengthy after literal translation, which, although adequate, threatened comprehensibility and ultimately validity. Item subordinating was needed to solve this problem.

Although this step was important to retrieve a comprehensive starting version equivalent to the source, our process has shown that an adaptation needs additional steps to successfully transfer a survey to a different cultural context. This is even more important in the case of patient self-reported measures that assess normative constructs, such as for the study of integrated care. As the respondents’ feedback has shown, although the cultures of the US and the Netherlands are relatively similar [25], considerable differences exist between integrated care perceived by patients in the Netherlands as opposed to the US. These differences relate to the health care context and are structural and cultural in nature, requiring adaptations of the survey content. As for cultural elements, Dutch patients perceived the word ‘instructions’ as patronizing and preferred the word ‘advice’. Also, Dutch patients with chronic disease experienced questions about instructions for the medication intake as redundant because they were familiar with this medication and did not need repetitive explanations. As for structural elements, questions about care outside regular office hours were not applicable to the Dutch health care system and needed adaptation because patients are typically referred to the general practice service.

As these examples show, over-reliance on the equivalence approach ensures formal congruence of the ‘signifiers’ (words and sentences) but misses possible contextual differences which affect their ‘signified’, hence perceived meaning [26]. Thus, if one is looking for a sheer instrument translation, equivalence methods such as a forward and backward translation will serve the purpose. For a culturally appropriate adaptation, however, the equivalence approach results in only a starting version. Contextualization related methods are needed to adapt the survey to specificities of the context in which the survey will be administered. We therefore advise researchers who want to introduce an instrument to a new cultural context to combine equivalence and contextualization methods. Yet, the proportion of methods from each approach might depend on the context for which an instrument is adapted and its difference with the source context. Future research should investigate the extent to which differences across contexts influence the mix of methods between approaches. Doing so could advance our understanding of requirements for an efficient translation process in which each steps adds value to the translation [13]. Translation and cultural adaptation processes are costly and time-consuming [11], and hence should be designed to provide meaningful contribution to the translation outcome. Hofstede’s classifications of national cultures [25] is one framework that could help explain how differences in contexts relate to the necessity for either equivalence or contextualization approaches. According to this framework, national cultures can be compared across different dimensions. Johnson and colleagues [27] have shown how differences in Hofstede’s dimensions relate to differences in response behaviour. Cultures with high power distance, that is the extent to which members of a society accept that power is distributed unequally [28], are associated with significantly higher extreme responding and significantly lower tendency for acquiescence. These findings illuminate how this framework could be useful in assessing distance in cultural values between the context in which an instrument is developed and the context to which the instrument is transferred. Understanding these differences would help with choosing the relative proportion of equivalence and contextualization-related approaches. Equivalence approaches may suffice for transferring instruments across countries with relatively similar scores on the Hofstede’s culture dimensions. Larger differences may require more contextualization-related approaches. Translation approaches should start with an assessment of similarities and differences in contexts before choosing approaches to perform the transfer.

Through this study we were able to show that it is possible to transfer instruments that assess the patient-perceived level of integrated care across countries and cultural contexts. In doing so, we advanced current insights about how different approaches impact on the translation outcome in providing in-depth results of our translation process and results. Our results are promising, but more research is needed to advance cross-cultural research in the field of integrated care. For example, this study focused on between-country differences. The influences of within-country differences, which result from increasingly diversifying cultures inside countries [29], have yet to be examined. Also, differences in context between the US and the Netherlands were relatively small [28]. Usability of equivalence- and contextualization-related approaches should be assessed in translation projects for countries with larger differences. Additionally, this study focused on assessing the interpretative equivalence of survey items, namely the extent to which concepts are similar or different across contexts through cognitive interviews. However, Johnson and colleagues [27] have shown that respondents from different cultures do not only differ in their perception of the constructs but also in the degree to which they perceive that needs are fulfilled. As described in the method section, we aimed to achieve procedural equivalence during the translation and adaptation process of the Dutch PPIC survey and added questions that ask about respondents’ needs for a certain service to assess these preferences. A next step is to further explore results related to procedural equivalence and to determine validity of the Dutch PPIC survey through psychometric testing. These analyses are currently underway based on a study that was performed in four primary care centres in the Netherlands.

## Conclusion

Both equivalence and contextualization approaches contributed significantly to the translation and cultural adaptation of the PPIC survey, supporting the need for methods from both approaches when preparing an instrument for cross-cultural use. However, the required mix of approaches might depend on the difference between contexts, the nature of the survey and the purpose for which the survey is translated. The results retrieved by combining methods from both approaches support the applicability of the PPIC survey to measure integrated care in the Netherlands. Although alterations were needed, patients recognized that the PPIC survey covers crucial points of their care perception and confirmed the need for an assessment of these points to improve care experience.
